# AOAC-OMA/MicroVal Harmonized Validation of Peel Plate^TM^ EB (*Enterobacteriaceae* Bacteria), First Action 2018.05

**DOI:** 10.1093/jaoacint/qsaa067

**Published:** 2020-06-17

**Authors:** Robert S Salter, Gregory W Durbin, Denisse Martinez, Patrick Bird, Benjamin Bastin, Erin Crowley

**Affiliations:** 1 Charm Sciences, Inc, 659 Andover St, Lawrence, MA 01843, USA; 2 Q Laboratories, Inc, 1400 Harrison Ave, Cincinnati, OH 45214, USA

## Abstract

**Background:**

Peel Plate^TM^  *Enterobacteriaceae* Bacteria (EB) is dried selective media on a 47 mm plastic plate that produces enzyme substrate colored colonies on rehydration and incubation for 24 h and up to 48 h at 37 ± 1°C.

**Purpose:**

The method validation compared quantification of EB to reference methods ISO 21528:2017 Parts 1 and 2.

**Methods:**

Matrixes compared were whole milk, skim powdered milk, vanilla ice cream, butter, infant formulas (soy- and dairy-based), infant cereals ± probiotic, environmental sponge swab of stainless steel surface, and poultry carcass rinse with two different peptone buffers.

**Results:**

In inclusivity and exclusivity studies, the method detected 54 of 54 EB strains and did not detect 30 of 30 non-EB strains. In matrix studies, the claimed foods were tested at three contamination levels using paired analysis between the reference and Peel Plate EB methods. Colony-forming units per gram or mL [CFU/g (mL)] were log_10_ transformed for statistical analysis. The candidate method and reference method were shown to be equivalent by the performance requirement of all 95% confidence intervals on mean difference falling between −0.5 and +0.5 log_10_ CFU/g (mL). An international collaborative study with dried infant formula spiked with *Cronobacter sakazakii* at log_10_ CFU/g (mL) 1.05, 2.31, and 3.21 levels, produced method differences −0.16, 0.15, and 0.18 log_10_ CFU/g (mL) with repeatabilities (*r*) = 0.33, 0.20, and 0.12 log_10_ CFU/g (mL) and reproducibilities (*R*) = 0.45, 0.26, and 0.18 log_10_ CFU/g (mL).

**Conclusions:**

Based on these evaluations, the candidate method is considered equivalent to the reference methods at both the 24 h and 48 h incubation periods at 37 ± 1°C.

**Highlights:**

Ready to use Enterobacteriaceae method equivalent to ISO-21528:2017 Parts 1 and 2; EB test colored colonies at 37°C for 24 h are equivalent at 48 h incubation; Singlet determined CFU/mL are statistically the same as duplicate average results; EB test validated for infant formula and dairy products including with probiotics; EB test for environmental surfaces and poultry carcass rinses using peptone buffers.

The *Enterobacteriaceae* is a family of Gram-negative, nonspore-forming *bacilli* bacteria and is one of the most important groups of bacteria known that are found in soil and water, as well as in plants and in animals (both vertebrates and invertebrates). They may be motile or nonmotile, depending on species. They are aerobic or facultatively anaerobic in growth and tend to inhabit the gastrointestinal tract.

Among the most notable foodborne pathogens and spoilage organisms are *Escherichia, Salmonella, Enterobacter, Klebsiella, Citrobacter, Cronobacter, Shigella,* and *Yersinia*. Methods for the detection and enumeration of *Enterobacteriaceae* have changed very little since they were first introduced and many still rely on the growth of the bacterium in selective media along with the use of carbohydrates as an energy source (1). Because *Enterobacteriaceae* are used so frequently by the food industry, there are needs for simple, low cost, ready-to-use methods for testing. Peel Plate EB is a simple method to detect and quantify *Enterobacteriaceae* in foods which is studied and validated in this work.

In the study, the target organisms are bacteria in the family *Enterobacteriaceae* that comprise a broad number of Gram-negative bacteria. Performance testing of heat-processed milk, dairy products, infant formula, cereals, stainless-steel surfaces, and chicken carcass rinses are not statistically different between candidate and reference methods. Statistical difference is determined from CFU/mL results log_10_ transformed and all 95% confidence intervals on mean difference between candidate and reference methods falling between −0.5 and +0.5 log_10_ CFU/g (mL) (2–4). Peel Plate EB is the candidate method and reference methods are ISO 21528-1:2017 Microbiology of the food chain—Horizontal method for detection and enumeration of *Enterobacteriaceae*—Part 1: Detection (5) and ISO 21528-2:2017 Part 2: Enumeration (6).

## AOAC *Official Method*^SM^ 2018.05 Enumeration of *Enterobacteriaceae in* Select Foods and Environmental Surfaces by Peel Plate EB *First Action 2018*

[Applicable to the enumeration of *Enterobacteriaceae* from pasteurized whole milk, butter, nonfat dry milk, vanilla ice cream, powdered and liquid infant formula (milk-based) containing probiotic, nonprobiotic liquid infant formula (soy-based), infant cereal with probiotic, infant rice cereal without probiotic, chicken carcass rinse with neutralized buffered peptone water, chicken carcass rinse with buffered peptone water, and stainless-steel surfaces.]

*Caution:* Perform tests with clean, washed, and gloved hands assuming potential pathogenic bacteria. Microbiological cultures and reagents should be collected into biohazardous bags and autoclaved. Dispose according to local, state, and federal regulations.

## A. Principle

Peel Plate^®^ EB test is used for the detection and enumeration of Enterobacteriaceae bacteria in food and environmental samples. The method is applicable for the determination of *Enterobacteriaceae* in samples when incubated at 37 ± 1°C for up to 24–48 h. All visible colonies, regardless of color, on the Peel Plate are to be considered an *Enterobacteriaceae*. The method limit of detection is 1 or greater CFU per milliliter or gram of test sample. The accurate quantitative range for *Enterobacteriaceae* is 1 to 150 CFU per plate.

The Peel Plate EB test is based on bile salt selective agar, glucose, and multiple colorimetric enzyme substrates to support growth and colormetrically identify the growth of the family of *Enterobacteriaceae* bacteria. The media also contains gelling and wicking agents which absorb and diffuse the sample.

## B. Apparatus

- *Peel Plate EB*.—Cat. Nos. PP-EB-100K (100 Peel Plate EB tests) and PP-EB-1000K (1000 Peel Plate EB tests). (1) *Test kit components*.—Two foil bags containing 50 Peel Plate EB each with blue indicator desiccants (Charm Sciences, Inc., Lawrence, MA, USA).
- *Pipet tips*.—1 mL.
- *Pipettor*.—1 mL.
- *Incubator.—*37 ± 1°C depending on test matrix.
- *Light box.—*For back illuminating and counting plates.
- *Magnifying glass.—*2× or 4× for examining plates.
- *Stomacher.—*Seward 400 paddle type, or equivalent.

## C. Reagents

- *Butterfield’s phosphate buffered dilution water (BPBDW)*.—Buffer KH_2_PO_4_ (34 g to 500 mL) with distilled (DI) or reverse osmosis (RO) water and adjust pH to 7.2 with 1 N NaOH. Bring final volume to 1 L with DI or RO water. Add 99 mL to dilution bottles and sterilize for 15 min at 121°C. Store in refrigerator. Or purchased, e.g., Weber Scientific (Hamilton, NJ, USA) Item No. 3127-14, or equivalent.
- *Buffered peptone water (BPW)*.—Peptone 10 g, sodium chloride 5 g, disodium phosphate 3.5 g, monopotassium phosphate 1.5 g, DI water 1 L. Add 99 mL to dilution bottles and sterilize for 15 min at 121°C. Store in refrigerator. Final pH 7.2 ± 0.2.
- *Neutralizing buffered peptone water* (*n-BPW*).—Buffered peptone 20.0 g, soy lecithin 7 g, sodium thiosulfate 1 g, microbiologically suitable (MS) water 1 L, sodium bicarbonate 12.5 g, pH 7.7 ± 0.5 at 25°C.

## D. General Preparation

- Observe Good Laboratory Practices for microbial testing. Avoid specimen contamination.
- Test on a level surface, in a clean area, and free of dust and blowing air.
- Avoid hand contact with test samples and Peel Plate EB medium.
- Log serially dilute sample into BPW, Butterfield’s, or MS water to obtain the countable range 1–150 CFU/plate or test multiple dilutions to attain the countable range.

## E. Sample Preparation

- *Foods*.—(1) Add 25 g (25 mL if already liquid) of food (infant formula, butter, milk, ice cream, milk powder) to 225 mL dilution buffer (BPW following ISO method), stomach/homogenize for 1–2 min, and let settle 1 min. Following homogenization, perform 1:10 serial dilutions in dilution buffer to the desired concentration. (2) For cereal, add 25 g to 1225 mL dilution buffer, stomach/homogenize for 1–2 min, and let settle 1 min. (3) Continue to dilute 10 mL of prior dilution in 90 mL dilution blank to reach countable range (1 to 150 CFU/plate). Other volume/volume dilution schemes are acceptable.
- *Surfaces*.—The sampling protocol followed ISO 18593 (7). Sample stainless-steel surfaces by rehydrating a sponge with 25 mL BPW, rinsing aseptically, swabbing a 100 cm^2^ surface, adding the sponge to buffer, and stomaching for 1–2 min.
- *Chicken carcasses*.—The sampling protocol followed FSIS Directives for chicken carcass (8, 9). Add 400 mL BPW or n-BPW to a bag with a chicken carcass, seal and shake bag for 1 min (10). Collect 100 mL rinse for testing.

## F. Method Procedure

- Place Peel Plate onto a level surface. Apply pressure with fingers to the rear rectangular platform to keep plate flat.
- Lift cover vertically upwards completely exposing the dried media culture disc. Leave cover adhered to back of plate.
- While holding cover up, keep plate flat on surface, vertically dispense 1.0 mL of sample or sample dilution to the center of exposed Peel Plate disc. Expel pipet contents rapidly with even force and within 2 to 3 s. Sample will self-wick to the edges of the disc. It is acceptable to lift and rotate plate to swirl sample to edges when sample conditions interfere with wicking.
  1. In the case of cereal, five plates should be rehydrated per sample. Alternatively, 5 mL homogenized sample is added to one high-volume plate.
- Reapply the adhesive cover without wrinkling. Press cover around edges of plate to ensure proper seal.
- Incubate plates with adhesive cover down, clear side up.
  1. Incubate at 37 ± 1°C for 24 up to 48 h.
  2. Plates can stack by aligning the small and large footings. Stacking up to 20 will not affect plate heat transfer.

## G. Interpretation and Test Result Report

- At the end of the incubation period, observe plates for colonies by viewing through the clear side of the Peel Plate EB. Each colored spot, regardless of color, represents 1 CFU. The sum of spots is reported as the *Enterobacteriaceae* CFU/mL of the diluted sample. In the case of cereal, sum the colonies from all five plates or count all the colonies on the 5 mL plate.
- Multiply CFU/plate by dilution factor (reciprocal of dilution) to calculate CFU/mL (or CFU/g) of original sample.
  1. In the case of cereal, as 5 mL are enumerated, the homogenization dilution is 10 (5 mL of 1 to 50 dilution).
  2. In the case of surfaces, the count is per mL of buffer used to sample the surface. Multiply by buffer volume and divide by cm^2^ of surface tested to calculate counts/cm^2^.
  3. In case of poultry rinse, the count is per mL of buffer used to rinse carcass.
- In case of spreading bacteria, score 1 CFU for each count each dark centered focal point within the spread growth as a single colony. Blended colonies are scored as a single CFU.
- Counts of 1 to 150 CFU/plate are considered countable, while counts outside that range are considered estimates. Samples with results outside of countable range (>150 CFU/plate) can be diluted and retested.

## H. Confirmation

The Peel Plate EB method uses selective medium and enzyme substrates to detect *Enterobacteriaceae* without the need for confirmation steps. Although it is not necessary, it may be desired to confirm colonies on traditional selective medium. The cover may be lifted and colonies picked and streaked onto violet red bile agar with glucose (VRBAG) broth. To confirm *Enterobacteriaceae,* isolates should be tested for oxidase activity and stabbed into glucose agar containing bromocresol blue and covered with sterile immersion oil. Oxidase negative samples that acidify glucose agar to produce yellow stab are confirmed EB. *Enterobacteriaceae* confirmation procedures are described in ISO protocols (5, 6).

## Precollaborative Validation Study

The validation study was conducted under a harmonized MicroVal/AOAC *Official Methods of Analysis* (OMA) design. This utilized ISO reference methods for foods, when the method existed, and the *AOAC INTERNATIONAL Methods Committee Guidelines for Validation of Microbiological Methods for Food and Environmental Surfaces* (11).

Method developer studies were conducted in at Charm Sciences, Inc. (Lawrence, MA, USA) and included supplemental matrix data for additional claimed matrixes, product consistency and stability studies, and robustness testing.

The independent precollaborative laboratory study was conducted by Q Laboratories, Inc. (Cincinnati, OH, USA) and included the inclusivity/exclusivity studies and matrix studies for the claimed food/and or surface matrixes. Q Laboratories prepared samples and coordinated an international eleven laboratory collaborative study of powdered infant formula containing probiotic.

The testing laboratories were SGS Vanguard Sciences (North Sioux City, ND, USA); ALS Marshfield LLC (Marshfield, WI, USA); Nestlé Research Center (Lausanne, Switzerland); Covance Laboratories (Madison, WI, USA); Joint Institute for Food Safety and Applied Nutrition (College Park, MD, USA); Environmental and Occupational Health Microbiology Lab, University of Washington (Seattle, WA, USA); HiPP Croatia d.o.o (Glina, Croatia); Maxxam Analytics (Mississauga, ON, Canada); Maxxam Analytics (Burnaby, BC, Canada); and Teagasc (Cork, Ireland).

### Inclusivity and Exclusivity Studies

The inclusivity and exclusivity evaluations were conducted at Q Laboratories. All test materials required for the Peel Plate EB method were provided by Charm Sciences, Inc.

- *Methodology*.—For the inclusivity evaluation of the Peel Plate EB, 50 *Enterobacteriaceae* were cultured in BPW (ISO) broth at 37 ± 1°C for 24 ± 2 h. The 30 exclusivity organisms were cultured in brain heart infusion (BHI) broth at 37 ± 1°C for 24 ± 2 h. The inclusivity and exclusivity organisms were serially diluted in 0.1% BPW to approximately 100 CFU/mL. All samples were blind-coded and randomized and analyzed by the Peel Plate EB method and ISO 21528-2. One milliliter of each culture was plated in duplicate. All plates were incubated at 37 ± 1°C for 24 and 48 h. Colonies were enumerated.
- *Results and Discussion*.—Tables 1 and 2 show details of the inclusivity/exclusivity bacterial study strains, respectively. Table 1 demonstrates that of 54 *Enterobacteriaceae* inclusivity isolates evaluated, 54 were correctly detected with enumerated values similar to the ISO method. Shown in Table 2, of the 30 exclusivity strains evaluated, 30 were correctly excluded by both the reference and candidate methods.

**Table 1.** Detailed results of the inclusivity evaluation

No.	Genus	Species	Source	Origin	Peel Plate EB24 h, CFU/mL	Peel Plate EB48 h, CFU/mL	ISO 21528-2, CFU/mL
1	*Citrobacter*	*amalonaticus*	ATCC^a^ 25405	Feces	14	14	13
2	*Citrobacter*	*koseri*	ATCC 27156	NA^b^	12	12	18
3	*Citrobacter*	*braakii*	ATCC 43162	Clinical isolate, California	16	16	20
4	*Citrobacter*	*farmeri*	ATCC 51633	Human feces	23	23	22
5	*Citrobacter*	*freundii*	QL^c^ 100813-2A	Sliced deli meat (turkey)	35	35	29
6	*Cronobacter*	*dublinensis*	DSM^d^ 18706	Infant formula	28	28	25
7	*Cronobacter*	*condimenti*	DSM 27966	Infant formula	36	36	30
8	*Cronobacter*	*helveticus*	CCUG^e^ 66106	Product industry	44	44	37
9	*Cronobacter*	*malonaticus*	CCUG 28859	Formula	27	27	29
10	*Cronobacter*	*muytjensii*	DSM 21870	Product industry	49	49	41
11	*Cronobacter*	*pulveris*	DSM 19145	Product industry	62	62	55
12	*Cronobacter*	*sakazakii*	CCUG 28863	Human cerebrospinal fluid	21	21	19
13	*Edwardsiella*	*tarda*	ATCC 15947	Feces, human	90	90	80
14	*Enterobacter*	*aerogenes*	ATCC 35029	NA	80	80	70
15	*Enterobacter*	*amnigenus*	ATCC 51816	Milk, Minnesota	110	110	90
16	*Enterobacter*	*cancerogenus*	QL 11010-2	Bottled water	42	42	37
17	*Enterobacter*	*cloacae*	NBRC^f^ 13536	NA	60	60	53
18	*Enterobacter*	*gergoviae*	ATCC 33028	Urine, France	54	54	49
19	*Escherichia*	*coli*	ATCC 8739	Feces	140	140	130
20	*Escherichia*	*vulneris*	ATCC 29943	Human wound	150	150	140
21	*Escherichia*	*fergusonii*	ATCC 35469	Feces, human	120	120	130
22	*Escherichia*	*hermannii*	ATCC 33651	Arm wound	80	80	70
23	*Shimwellia*	*blattae*	ATCC 29907	Hindgut of cockroach	50	50	40
24	*Hafnia*	*alvei*	ATCC 51815	Milk, Minnesota	80	80	60
25	*Klebsiella*	*pneumoniae*	ATCC 11296	NA	90	90	70
26	*Klebsiella*	*oxytoca*	ATCC 43165	Clinical isolate	40	40	40
27	*Kluyvera*	*intermedia*	ATCC 33110	Surface water	50	50	60
28	*Pantoea*	*agglomerans*	ATCC^a^ 19552	Sewage	70	70	100
29	*Morganella*	*morganii*	ATCC 25829	Human	80	80	90
30	*Proteus*	*hauseri*	ATCC 13315	Human feces	80	80	80
31	*Proteus*	*mirabilis*	ATCC 9240	Unknown	160	160	140
32	*Proteus*	*vulgaris*	ATCC 6380	Clinical isolate	150	150	130
33	*Providencia*	*rettgeri*	ATCC 14505	NA	150	150	130
34	*Providencia*	*stuartii*	QL^c^ 11007-5	Environmental isolate	90	90	100
35	*Rahnella*	*aquatilis*	ATCC 55046	Soil, Wisconsin	80	80	80
36	*Salmonella*	*bongori*	NCTC^d^ 10946	Amphibian; frog	80	80	70
37	*Salmonella*	*enterica* Anatum	ATCC 9270	Pork liver, Chicago, IL, USA	100	100	110
38	*Salmonella*	*enterica* subsp. *Arizonae*	QL 11007-4	Veterinary	130	130	110
39	*Salmonella*	*enterica* Choleraesuis	ATCC 53000	X-ray-induced mutant	70	70	70
40	*Salmonella*	*enterica* subsp. *diarizona*	QL 011414.1	Environmental isolate	41	41	37
41	*Salmonella*	*enterica* subsp. *diarizonae*	ATCCBAA-639	Feces, human	90	90	90
42	*Salmonella*	*enterica* subsp. *enterica* Infantis	ATCC 51741	Pasta	210	210	170
43	*Salmonella*	*enterica* Newport	ATCC 6962	Food poisoning	120	120	100
44	*Salmonella*	*enterica* Pullorum	ATCC 13036	Egg	100	100	90
45	*Salmonella*	*enterica* subsp. *enterica Typhimurium*	ATCC 14028	Tissue, animal	110	110	120
46	*Salmonella*	*enterica* subsp. *houtenae* Enteritidis	ATCC 13076	NA	100	100	90
47	*Serratia*	*liquefacians*	ATCC 27592	Milk, Cork, Ireland	110	110	110
48	*Serratia*	*marcescens*	ATCC 8100	NA	120	120	130
49	*Siccibacter*	*turicensis*	CCUG^e^ 54945	NA	32	32	27
50	*Yersinia*	*enterocolitica*	ATCC 49397	Clinical specimen	29	29	31
51	*Salmonella*	*enterica* subsp. *indica*	NCTC 10458	Desiccated coconut	40	40	30
52	*Salmonella*	*Enterica* houtenae	ATCC 15783	*Boa constrictor*, NL	130	130	110
53	*Salmonella*	*enterica* subsp. salamae	QL 02415	Dry pet food	140	140	100
54	*Shigella*	*boydii*	ATCC 9207	Pork liver	150	150	130

a ATCC = American Type Culture Collection.

b NA = Not available.

c QL = Q Laboratories Culture Collection.

d NCTC = National Collection Type Cultures.

e CCUG = University of Goteborg Culture Collection.

f NBRC = Nite Biological Resource Center.

**Table 2.** Detailed results of the exclusivity evaluation

No.	Genus	Species	Source	Origin	Peel Plate EB24 h, CFU/mL	Peel Plate EB48 h, CFU/mL	ISO 21528-2, CFU/mL
1	*Acinetobacter*	*baumanii*	ATCC^a^ 19606	Urine	<1	<1	<1
2	*Aeromonas*	*viridans*	QL^b^ 17041-8	Raw milk isolate	<1	<1	<1
3	*Alcaligenes*	*faecalis*	ATCC 8750	NA^c^	<1	<1	<1
4	*Bacillus*	*cereus*	ATCC 6464	Soil	<1	<1	<1
5	*Bacillus*	*subtilis*	ATCC 6633	NA	<1	<1	<1
6	*Bordetella*	*bronchiseptica*	ATCC 10580	Lung of dog	<1	<1	<1
7	*Brochothrix*	*thermosphacta*	ATCC 11509	Animal-derived foodstuff	<1	<1	<1
8	*Enterococcus*	*durans*	ATCC 19432	NA	<1	<1	<1
9	*Enterococcus*	*faecalis*	ATCC 29212	Urine	<1	<1	<1
10	*Enterococcus*	*faecium*	ATCC 51559	Clinical isolate	<1	<1	<1
11	*Enterococcus*	*hirae*	ATCC 8043	NA	<1	<1	<1
12	*Haemophilus*	*influenzae*	ATCC 19418	NA	<1	<1	<1
13	*Kurthia*	*gibsonii*	ATCC 43195	Meat	<1	<1	<1
14	*Kurthia*	*zopfii*	ATCC 10538	NA	<1	<1	<1
15	*Leuconostoc*	*mesenteroides*	ATCC 8293	Fermenting olives	<1	<1	<1
16	*Listeria*	*innocua*	ATCC 33090	Cow brain	<1	<1	<1
17	*Listeria*	*ivanovii*	ATCC BAA-139	Washing water	<1	<1	<1
18	*Listeria*	*monocytogenes*	ATCC 7644	Human isolate	<1	<1	<1
19	*Listeria*	*seeligeri*	ATCC 11289	Human feces	<1	<1	<1
20	*Listeria*	*welshimeri*	ATCC 43549	Soil	<1	<1	<1
21	*Micrococcus*	*luteus*	ATCC 10240	Air	<1	<1	<1
22	*Pseudomonas*	*alcaligenes*	ATCC 14909	Swimming pool water	<1	<1	<1
23	*Pseudomonas*	*extremorientalis*	QL 17041-1	Raw milk isolate	<1	<1	<1
24	*Pseudomonas*	*fluorescens*	QL 17041-3	Raw milk isolate	<1	<1	<1
25	*Staphylococcus*	*hominis*	ATCC 27844	Human skin	<1	<1	<1
26	*Staphylococcus*	*aureus*	ATCC 6538	Human lesion	<1	<1	<1
27	*Streptococcus*	*pneumoniae*	ATCC 6302	NA	<1	<1	<1
28	*Streptococcus*	*pyogenes*	ATCC 19615	Pharynx of child following sore throat	<1	<1	<1
29	*Vibrio*	*parahaemolyticus*	ATCC 17802	Shirasu food poisoning	<1	<1	<1
30	*Vibrio*	*vulnificus*	QL 021111A	Seafood product	<1	<1	<1

a ATCC = American Type Culture Collection.

b QL = Q Laboratories Culture Collection.

c NA = Not available.

### Precollaborative Matrix Study

Precollaborative matrix studies were conducted at Q Laboratories and at Charm Sciences, Inc. In these studies, each claimed matrix was evaluated naturally and at three contamination levels. The study outline adhered to Appendix J of the *Official Methods of Analysis of AOAC INTERNATIONAL* (11). Each food matrix was purchased from a local distributor, and prescreened for natural contamination of the target analyte by the ISO 21528-2:2017 reference method. Following the screening, each matrix tested by the validation laboratory was inoculated with a different strain of *Enterobacteriaceae* as indicated in Table 3. Additional matrixes were performed by Charm Sciences, Inc.

**Table 3.** Summary of categories, types, items, strains, and inoculation levels for the matrix study

Food category	Food type	Food item	Replicates/test portion size	Inoculating organism (culture conditions)	Achieved contamination levels^a^, CFU/g (mL)
Heat-processed milk and dairy products	Pasteurized milk-based products	3.25% Pasteurized whole milk	5 × 25 g	*Enterobacter amnigenus* (ATCC^b^ 51816; heat-stressed)	10–100
			5 × 25 g		100–5000
			5 × 25 g		5000–100 000
	Dry milk powder	Milk powder	5 × 25 g	*Hafnia alvei* (ATCC 51815; lyophilized)	10–100
			5 × 25 g		100–5000
			5 × 25 g		5000–100 000
Infant formula and infant cereals	Infant formula (milk-based) with probiotic	Infant formula with probiotic	5 × 25 g	*Cronobacter sakazakii* (CCUG^c^ 28863; lyophilized)	10–100
			5 × 25 g		100–5000
			5 × 25 g		5000–100 000
	Infant cereal with probiotic	Infant cereal with probiotic	5 × 25 g	*Escherichia coli* (ATCC 25922; lyophilized)	10–100
			5 × 25 g		100–5000
			5 × 25 g		5000–100 000
Environmental surfaces	Stainless-steel food contact surface	NA^d^	4 × 4 in. sq.	Salmonella enterica subsp. enterica Typhimurium (ATCC 14028)	10–100
			4 × 4 in. sq.		100–5000
			4 × 4 in. sq.		5000–100 000
In-process sample	Carcass rinse	Chicken	Carcass	Natural contamination	10–100
			Carcass		100–5000
			Carcass		5000–100 000

a The uninoculated and the low contamination levels were blind-coded and evaluated by ISO 21528–1:2017 reference method. The medium and high contamination levels were blind-coded and evaluated by the ISO 21528-2:2017 reference method.

b ATCC = American Type Culture Collection.

c CCUG = University of Goteborg Culture Collection.

d NA = Not available.

- *Methodology*.—The precollaborative comparison study consisted of evaluating a total of 20 paired sample replicates for 3.25% pasteurized whole milk, nonfat dry milk powder, infant formula with probiotic, stainless steel, and chicken carcass rinse. In the case of infant cereal with probiotic, the candidate method called for a greater dilution in preparation than the reference method, so unpaired samples were used. Within each food matrix sample set there was an uninoculated level and three target inoculation ranges: five uninoculated samples (0 CFU/mL), five low-level inoculated samples (10–100 CFU/mL), five medium-level inoculated samples (100–5000 CFU/mL), and five high-level inoculated samples (5000–100 000 CFU/mL). In all matrix studies except chicken rinse, which had natural contamination, *Enterobacteriaceae* strains shown in Table 3 from cultures were spiked and acclimated in products for 48 to 72 hours before testing. The acclimated material was quantified using the ISO method and then used for creating fortification levels. Each inoculum was prepared by transferring a single colony from trypticase soy agar with 5% sheep blood (SBA) into BHI broth and incubating the culture at 35 ± 2°C for 24 ± 2 h. Following incubation, the culture was diluted to a target level using BHI as the diluent. For each inoculated food matrix, bulk portions were spiked and blended in large, sterile stainless-steel containers. Sterile spatulas were used to mix the bulk portions to ensure the inoculum was evenly distributed throughout the matrix. The 3.25% pasteurized whole milk was held for 48–72 h at refrigerated temperature (2–8°C) prior to analysis to allow time for the organism to equilibrate within the sample. For nonfat dry milk powder, infant formula with probiotic, and infant cereal with probiotic, a lyophilized inoculum was used to inoculate a bulk lot of each matrix and was then homogenized and held at ambient temperature (20–25°C) for 2 weeks. Prior to inoculation of 3.25% pasteurized whole milk, the broth culture inoculum was heat stressed in a water bath for 10 ± 1 min at 50 ± 1°C. The degree of injury of each culture was estimated by plating an aliquot of diluted culture onto violet red bile (VRB) agar and tryptic soy agar (TSA). The agars were incubated at 35 ± 1°C for 24 ± 2 h and the colonies enumerated. The percent of injury was estimated:
  $$(1-\frac{n_{\mathrm{select}}}{n_{\mathrm{nonselect}}})x100$$

where *n*_select_ = number of colonies on selective agar and *n*_nonselect_ = number of colonies on nonselective agar.

Stainless-steel and sealed concrete surfaces were evaluated after artificial contamination. Each test portion area (4 × 4 in.) was evenly inoculated with 250 µL *Salmonella enterica* subsp. *enterica* serovar Typhimurium ATCC (American Type Culture Collection, Manassas, VA, USA) 14028 diluted in BHI and allowed to dry for 16–24 h at ambient temperature (20–25°C). The environmental surface was sampled using horizontal and vertical sweeping motions. Sampling sponges were held for a minimum of 2 h at ambient temperature prior to analysis. To determine the inoculation level for the environmental surface, aliquots of each inoculating organism was plated in duplicate onto TSA and enumerated.

Chicken carcass rinse was positive for natural contamination *Enterobacteriaceae*. Different lots of the matrix were purchased and screened to identify varying contamination levels. Lots were then mixed to produce three levels of contamination. The chicken carcass rinse was evaluated using naturally occurring *Enterobacteriaceae*. Within these sample sets, there were five replicates evaluated at a low contamination level targeting 10–100 CFU/g, five replicates evaluated at a medium contamination level targeting 100–1000 CFU/g, and five replicates evaluated at a high contamination level targeting 1000–10 000 CFU/g.

(**b**) *ISO 21528-1:2017 (low levels of contamination; <100 CFU/g or mL)*.—Using the paired test portions, 25 g test portions were combined with 225 mL BPW (ISO) and homogenized by stomaching for 2 min ± 15 s. From each sample, 10 mL of the initial 1:10 dilution was transferred into three separate test tubes (10^−1^). A 1 mL transfer of the initial 1:10 dilution was transferred into three test tubes (10^−2^) containing 9 mL BPW (6). One additional dilution was performed by transferring 1 mL of the 10^−2^ dilution into each of the three test tubes (10^−3^) containing 9 mL BPW (5). All tubes were incubated at 37 ± 1°C for 18 ± 2 h. Following incubation of the tubes, all tubes were streaked onto violet red bile agar with glucose (VRBAG) agar and incubated at 37 ± 1°C for 24 ± 2 h. After incubation, all plates were examined for typical *Enterobacteriaceae* colony morphology. Up to five characteristic colonies were streaked to TSA and incubated at 37 ± 1°C for 24 ± 2 h. From an isolated colony from each of the TSA plates, a spot oxidase test was performed. For each oxidase negative colony, a stab to Glucose OF Medium and an overlay of sterile mineral oil was added. All Glucose OF Medium tubes were incubated at 37 ± 1°C for 24 ± 2 h. If a yellow color developed, the reaction was considered positive. The Most Probable Number (MPN) levels and confidence limits were determined by Table B.5 in ISO 7218:2007 (E) (12).
(**c**) *ISO 21528-2:2017 (medium to high levels of contamination; >100 CFU/g or mL).—*Using the paired test portions, a 25 g test portion was combined with 225 mL of 0.1% BPW and homogenized by stomaching for 2 min ± 15 s. Further 1:10 serial dilutions were conducted in order to achieve the desired target concentrations. A 1 mL aliquot of each dilution was plated in duplicate and 10 mL of tempered VRBAG agar was added to each plate. After the plates were completely solidified, an overlay of approximately 8–15 mL VRBAG was added to each plate. All plates were incubated at 37 ± 1°C for 24 ± 2 h. Following incubation, plates containing <150 pink to red and purple CFU were enumerated. The average CFU of the duplicate plates was recorded and multiplied by the dilution factor (reciprocal of dilution) and reported as total *Enterobacteriaceae* CFU/g or mL. Up to five typical colonies were streaked to TSA and incubated at 37 ± 1°C for 24 ± 2 h. A spot oxidase test was conducted for each plate, all oxidase negative colonies were stabbed into Glucose OF Medium with an overlay of sterile mineral oil. All Glucose OF Medium tubes were incubated at 37 ± 1°C for 24 ± 2 h. If a yellow color developed, the reaction was considered positive.
(**d**) *Peel Plate^®^ EB method.—*All matrixes were diluted according to the AOAC protocol as described previously in “Method Procedure.” After dilution, all test portions were plated following the Peel Plate EB method or in the case of cereal also the Peel Plate EBHV^®^ (high volume 5 mL) method.

**Table 5.** Peel Plate EB method (duplicate count) for *Enterobacteriaceae* at 24 h compared to ISO methods 21528-1 and 2

Matrix	Fortified micro-o rganisms (ATCC No.; % injury)	Contam. level	Candidate method		Reference method		Mean diff.^c^	95% CI^e^		*r* ^2h^
			Mean^a^	*s*_r_ ^b^	Mean^a^	*s*_r_ ^b^		LCL^f^	UCL^g^	
3.25% Pasteurized whole milk^i^	*Enterobacter amnigenus* (ATCC 51816; heat-stressed)	None	<0.1	NA^j^	<0.1	NA	NA	NA	NA	0.99
		Low	1.02	0.19	1.02	0.29	−0.00	−0.17	0.16	
		Medium	3.49	0.15	3.43	0.09	0.06	−0.11	0.22	
		High	4.18	0.10	4.20	0.14	−0.02	−0.13	0.09	
Nonfat dry milk powder^i^	*Hafnia alvei* (ATCC 51815; lyophilized)	None	<0.1	NA	<0.1	NA	NA	NA	NA	0.99
		Low	1.82	0.15	1.90	0.20	−0.08	−0.17	0.01	
		Medium	3.59	0.06	3.50	0.11	0.09	−0.01	0.19	
		High	4.99	0.17	4.89	0.08	0.10	−0.17	0.36	
Infant formula with probiotic^i^	*Cronobacter sakazakii* (CCUG^2^ 28863; lyophilized)	None	<0.1	NA	<0.1	NA	NA	NA	NA	0.99
		Low	1.78	0.11	1.67	0.23	0.11	−0.17	0.38	
		Medium	3.64	0.06	3.61	0.06	0.03	−0.00	0.06	
		High	4.89	0.08	4.83	0.07	0.06	−0.04	0.16	
Infant cereal with probiotic^i^	*Escherichia coli* (ATCC 25922; lyophilized)	None	<0.1	NA	<0.1	NA	NA	NA	NA	0.99
		Low	2.20	0.12	2.22	0.15	−0.02	−0.09	0.06	
		Medium	3.18	0.10	3.20	0.10	−0.02	−0.13	0.09	
		High	4.95	0.10	4.89	0.15	0.06	−0.08	0.21	
Sponge sample of stainless steel^i^	*Salmonella* Typhimurium (ATCC 14028)	None	<0.1	NA	<0.1	NA	NA	NA	NA	1.00
		Low	1.70	0.07	1.81	0.21	−0.11	−0.33	0.11	
		Medium	3.25	0.03	3.25	0.05	0.00	−0.07	0.07	
		High	4.63	0.01	4.60	0.02	0.03	0.00	0.06	
Chicken rinse in n-BPW^i^	Natural contamination	Low	1.15	0.09	1.26	0.11	−0.11	−0.22	0.01	1.00
		Medium	2.45	0.03	2.37	0.03	0.08	0.07	0.09	
		High	3.60	0.03	3.60	0.02	0.00	−0.03	0.03	
Unsalted butter	*Serratia marcescens* (ATCC 13880; 48% heat-stress injury)	None	<0.1	NA	<0.1	NA	NA	NA	NA	1.00
		Low	1.54	0.24	1.66	0.00	−0.12	−0.42	0.17	
		Medium	2.87	0.12	3.08	0.09	−0.21	−0.36	−0.05	
		High	5.46	0.20	5.53	0.09	−0.07	−0.26	0.11	
Vanilla ice cream	*Klebsiella oxytoca* (ATCC 700324; 42% heat-stress injury)	None	<0.1	NA	<0.1	NA	NA	NA	NA	1.00
		Low	1.49	0.27	1.55	0.15	−0.06	−0.30	0.17	
		Medium	4.91	0.06	5.04	0.04	−0.13	−0.22	−0.04	
		High	5.46	0.12	5.56	0.20	−0.10	−0.35	0.15	
Soy infant formula	*Enterobacter aerogenes* (ATCC 13048; 20% heat-stress injury)	None	<0.1	NA	<0.1	NA	NA	NA	NA	1.00
		Low	1.25	0.32	0.98	0.42	0.27	0.07	0.46	
		Medium	3.04	0.02	3.06	0.03	−0.02	−0.05	0.02	
		High	3.99	0.04	3.94	0.04	0.05	−0.01	0.12	
Chicken rinse in BPW	Natural contamination	Low	1.84	0.35	1.94	0.44	−0.10	−0.27	0.07	0.99
		Medium	2.46	0.08	2.43	0.29	0.03	−0.30	0.36	
		High	3.46	0.32	3.52	0.52	−0.06	−0.41	0.29	
Rice infant cereal	*Citrobacter freundii* (ATCC 8090; lyophilized)	None	<0.1	NA	<0.1	NA	NA	NA	NA	1.00
		Low	1.42	0.41	1.63	0.27	−0.21	−0.49	0.08	
		Medium	3.49	0.07	3.56	0.14	−0.07	−0.18	0.05	
		High	4.45	0.09	4.56	0.06	−0.11	−0.27	−0.04	

a Mean of five replicate portions, plated in duplicate, after logarithmic transformation: log_10_[CFU/g (mL) + (0.1)*f*].

b Repeatability standard deviation.

c Mean difference between the candidate and reference methods.

d Confidence interval.

e 95% Lower confidence limit for difference of means.

f 95% Upper confidence limit for difference of means.

g Square of correlation coefficient.

h Independent lab performed.

i NA = Not applicable.

Statistical analysis was conducted for each contamination level for each matrix evaluated comparing the Peel Plate EB method to the ISO reference method (2–4). Logarithmic transformations of the counts [CFU/g (mL)] were performed, and the difference of means, with 95% confidence intervals, between the candidate method and the reference method was determined for each contamination level. Mean difference and confidence intervals were calculated using the Independent Laboratory Study Workbook for Paired Method Analysis for Micro Testing (Version 1.0) supplied by the AOAC Research Institute (2). A mean difference between methods of <0.5 log_10_ CFU/g (mL) with a 95% confidence interval (CI) containing values between [−0.5 log_10_ CFU/g (mL), 0.5 log_10_ CFU/g (mL)] was used as guidance to determine statistically significant differences between two methods being compared. The repeatability (*s_r_*) of the Peel Plate EB and ISO reference methods were determined for each matrix.

(**e**) *Results and Discussion*.—Tables 4–7 are summary tables of evaluated matrixes, showing the spiked bacteria or natural contamination log_10_ CFU/g (mL) levels evaluated, and the resulting mean averages and *s*_r_ from five paired results between the Peel Plate EB and reference methods. The tables include mean differences associated between the candidate and reference with the confidence limits and correlation coefficient, *r*^2^, of the mean linear regression curve.

**Table 4.** Peel Plate EB method (singlet count) for *Enterobacteriaceae* at 24 h compared to ISO methods 21528-1 and 2

Matrix	Fortified micro- organisms (ATCC No.)	Contamination level	Candidate method		Reference method		Mean difference^c^	95% CI^e^		*r* ^2h^
			Mean^a^	*s*_r_ ^b^	Mean^a^	*s*_r_ ^b^		LCL^f^	UCL^g^	
3.25% Pasteurized whole milk^i^	*Enterobacter amnigenus (*ATCC^1^ 51816; heat-stressed)	None	<0.1	NA^j^	<0.1	NA	NA	NA	NA	
		Low	1.03	0.23	1.02	0.29	0.01	−0.20	0.23	0.99
		Medium	3.45	0.15	3.43	0.09	0.02	−0.11	0.15	
		High	4.18	0.12	4.20	0.14	−0.02	−0.13	0.09	
Nonfat dry milk powder^i^	*Hafnia alvei* (ATCC 51815; lyophilized)	None	<0.1	NA	<0.1	NA	NA	NA	NA	0.91
		Low	1.86	0.15	1.90	0.20	−0.04	−0.15	0.09	
		Medium	3.56	0.10	3.50	0.11	0.06	−0.03	0.14	
		High	3.96	0.14	4.89	0.08	0.07	−0.12	0.29	
Infant formula with probiotic^i^	*Cronobacter sakazakii* (CCUG^2^ 28863; lyophilized)	None	<0.1	NA	<0.1	NA	NA	NA	NA	1.00
		Low	1.77	0.14	1.67	0.23	0.10	−0.06	0.26	
		Medium	3.64	0.06	3.61	0.06	0.03	−0.03	0.08	
		High	4.84	0.10	4.83	0.07	0.01	−0.06	0.08	
Infant cereal with probiotic^i^	*Escherichia coli* (ATCC 25922; lyophilized)	None	<0.1	NA	<0.1	NA	NA	NA	NA	0.99
		Low	2.20	0.08	2.22	0.15	−0.02	−0.10	0.08	
		Medium	3.15	0.13	3.20	0.10	−0.05	−0.17	0.07	
		High	4.95	0.12	4.89	0.15	0.06	−0.13	0.24	
Sponge sample from stainless steel^i^	*Salmonella* Typhimurium (ATCC 14028)	None	<0.1	NA	<0.1	NA	NA	NA	NA	1.00
		Low	1.69	0.07	1.63	0.09	0.06	−0.01	0.13	
		Medium	3.24	0.06	3.25	0.09	−0.01	−0.12	0.10	
		High	4.64	0.03	4.63	0.05	0.01	−0.06	0.08	
Chicken rinse in n-BPW^i^	Natural contamination	Natural	1.18	0.09	1.15	0.11	0.03	−0.01	0.07	1.00
		Medium	2.48	0.04	2.47	0.03	0.01	−0.03	0.05	
		High	3.59	0.06	3.56	0.05	0.03	−0.08	0.14	
Unsalted butter	*Serratia marcescens* (ATCC 13880; 48% heat-stress injury)	None	<0.1	NA	<0.1	NA	NA	NA	Na	1.00
		Low	1.57	0.15	1.66	0.00	−0.09	−0.27	0.09	
		Medium	2.86	0.12	3.08	0.09	−0.22	−0.37	−0.05	
		High	5.47	0.11	5.46	0.20	0.01	−0.20	0.23	
Vanilla ice cream	*Klebsiella oxytoca* ATCC 700324 (42% heat stress injury)	None	<0.1	NA	<0.1	NA	NA	NA	NA	1.00
		Low	1.46	0.34	1.55	0.15	−0.09	−0.41	0.22	
		Medium	4.91	0.05	5.04	0.04	−0.13	−0.21	−0.05	
		High	5.50	0.14	5.56	0.20	−0.06	−0.30	0.19	
Soy infant formula	*Enterobacter aerogenes* (ATCC 13048; 20% heat-stress injury)	None	<0.1	NA	<0.1	NA	NA	NA	NA	1.00
		Low	1.28	0.40	0.98	0.42	0.30	0.13	0.47	
		Medium	3.05	0.02	3.06	0.03	−0.01	−0.06	0.05	
		High	4.00	0.04	3.94	0.04	0.06	−0.01	0.13	
Chicken rinse in BPW	Natural contamination	Low	1.75	0.38	1.94	0.44	−0.19	−0.34	−0.04	0.99
		Medium	2.40	0.15	2.43	0.29	−0.03	−0.38	0.32	
		High	3.45	0.33	3.48	0.48	−0.03	−0.36	0.31	
Rice infant cereal	*Citrobacter freundii* (ATCC 8090; lyophilized)	None	<0.1	NA	<0.1	NA	NA	NA	NA	1.00
		Low	1.39	0.38	1.63	0.27	−0.24	−0.41	−0.07	
		Medium	3.53	0.05	3.56	0.14	−0.03	−0.17	0.10	
		High	4.48	0.05	4.56	0.06	−0.08	−0.20	0.02	

a Mean of five replicate portions, candidate singlet result and reference plated in duplicate, after logarithmic transformation: log_10_[CFU/g (mL) + (0.1)*f*].

b Repeatability standard deviation.

c Mean difference between the candidate and reference methods.

d Confidence interval.

e 95% Lower confidence limit for difference of means.

f 95% Upper confidence limit for difference of means.

g Square of correlation coefficient.

h Independent laboratory performed.

i NA = Not applicable.

**Table 6.** Peel Plate EB method (singlet count) for *Enterobacteriaceae* at 48 h compared to ISO methods 21528-1 and 2

Matrix	Fortified micro-organisms (ATCC No.)	Contamination level	Candidate method		Reference method		Mean difference^c^	95% CI^e^		*r* ^2h^
			Mean^a^	*s*_r_ ^b^	Mean^a^	*s*_r_ ^b^		LCL^f^	UCL^g^	
3.25% Pasteurized whole milk^i^	*Enterobacter amnigenus* (ATCC 51816; heat-stressed)	None	<0.1	NA^j^	<0.1	NA	NA	NA	NA	0.99
		Low	1.04	0.24	1.02	0.29	0.02	−0.19	0.23	
		Medium	3.47	0.15	3.43	0.09	0.04	−0.10	0.17	
		High	4.18	0.12	4.20	0.14	−0.02	−0.13	0.09	
Nonfat dry milk powder^j^	*Hafnia alvei* (ATCC 51815; lyophilized)	None	<0.1	NA	<0.1	NA	NA	NA	NA	0.91
		Low	1.89	0.19	1.90	0.20	−0.01	−0.09	0.08	
		Medium	3.56	0.09	3.50	0.11	0.06	−0.03	0.14	
		High	4.99	0.15	4.89	0.08	0.10	−0.10	0.30	
Infant formula with probiotic^i^	*Cronobacter sakazakii* (CCUG^2^ 28863; lyophilized)	None	<0.1	NA	<0.1	NA	NA	NA	NA	1.00
		Low	1.77	0.14	1.67	0.23	0.10	−0.06	0.25	
		Medium	3.66	0.06	3.61	0.06	0.05	−0.01	0.10	
		High	4.86	0.12	4.83	0.07	0.03	−0.06	0.11	
Infant cereal with probiotic^i^	*Escherichia coli* (ATCC 25922; lyophilized)	None	<0.1	NA	<0.1	NA	NA	NA	NA	0.99
		Low	2.21	0.08	2.22	0.15	−0.01	−0.10	0.09	
		Medium	3.16	0.13	3.20	0.10	−0.04	−0.17	0.08	
		High	4.96	0.12	4.89	0.15	0.07	−0.14	0.27	
Sponge sample from stainless steel^i^	*Salmonella* Typhimurium (ATCC 14028)	None	<0.1	NA	<0.1	NA	NA	NA	NA	1.00
		Low	1.72	0.07	1.63	0.09	0.09	−0.09	0.24	
		Medium	3.26	0.06	3.25	0.09	0.01	−0.12	0.12	
		High	4.64	0.03	4.63	0.05	0.01	−0.05	0.08	
Chicken rinse in n-BPW^i^	Natural contamination	Low	1.19	0.08	1.15	0.11	0.04	−0.00	0.08	1.00
		Medium	2.49	0.04	2.47	0.03	0.02	−0.02	0.06	
		High	3.60	0.05	3.56	0.05	0.04	−0.07	0.14	
Unsalted butter	*Serratia marcescens* ATCC 13880 (48% heat-stress injury)	None	<0.1	NA	<0.1	NA	NA	NA	NA	1.00
		Low	1.61	0.18	1.66	0.00	−0.05	−0.27	0.16	
		Medium	2.87	0.12	3.08	0.09	−0.21	−0.37	−0.05	
		High	5.47	0.11	5.46	0.20	0.01	−0.20	0.23	
Vanilla ice cream	*Klebsiella oxytoca* (ATCC 700324; 42% heat-stress injury)	None	<0.1	NA	<0.1	NA	NA	NA	NA	1.00
		Low	1.56	0.38	1.55	0.15	0.01	−0.30	0.31	
		Medium	4.94	0.04	5.04	0.04	−0.10	−0.17	−0.04	
		High	5.51	0.15	5.56	0.20	−0.05	−0.30	0.21	
Soy infant formula	*Enterobacter aerogenes* (ATCC 13048; 20% heat-stress injury)	None	<0.1	NA	<0.1	NA	NA	NA	NA	1.00
		Low	1.29	0.42	0.98	0.42	0.31	0.14	0.48	
		Medium	3.05	0.02	3.06	0.03	−0.01	−0.06	0.05	
		High	4.00	0.04	3.94	0.04	0.06	−0.01	0.13	
Chicken rinse in BPW	Natural contamination	Low	1.74	0.37	1.94	0.44	−0.20	−0.38	−0.03	0.99
		Medium	2.40	0.14	2.43	0.29	−0.03	−0.32	0.26	
		High	3.44	0.33	3.48	0.48	−0.04	−0.31	0.22	
Rice infant cereal	*Citrobacter freundii* (ATCC 8090; lyophilized)	None	<0.1	NA	<0.1	NA	NA	NA	NA	1.00
		Low	1.42	0.38	1.63	0.27	−0.21	−0.37	−0.05	
		Medium	3.53	0.05	3.56	0.14	−0.03	−0.17	0.10	
		High	4.48	0.05	4.56	0.06	−0.08	−0.20	0.03	

a Mean of five replicate portions, candidate singlet result and reference plated in duplicate, after logarithmic transformation: log_10_[CFU/g (mL) + (0.1)*f*].

b Repeatability standard deviation.

c Mean difference between the candidate and reference methods.

d Confidence interval.

e 95% Lower confidence limit for difference of means.

f 95% Upper confidence limit for difference of means.

g Square of correlation coefficient.

h Independent lab performed.

i NA = Not applicable.

2 Culture Collection University of Gothenburg, SE.

**Table 7.** Peel Plate EB method (duplicate count) for *Enterobacteriaceae* at 48 h compared to ISO methods 21528-1 and 2

Matrix	Fortified micro- organisms (ATCC No.)	Contamination level	Candidate method		Reference method		Mean difference^c^	95% CI^e^		*r* ^2h^
			Mean^a^	*s*_r_ ^b^	Mean^a^	*s*_r_ ^b^		LCL^f^	UCL^g^	
3.25% Pasteurized whole milk^i^	*Enterobacter amnigenus* (ATCC^1^ 51816; heat-stressed)	None	<0.1	NA^j^	<0.1	NA	NA	NA	NA	0.99
		Low	1.03	0.20	1.02	0.29	0.01	−0.14	0.15	
		Medium	3.49	0.15	3.43	0.09	0.06	−0.11	0.22	
		High	4.19	0.11	4.20	0.14	−0.01	−0.12	0.10	
Nonfat dry milk powder^i^	*Hafnia alvei* (ATCC 51815; lyophilized)	None	<0.1	NA	<0.1	NA	NA	NA	NA	0.99
		Low	1.89	0.14	1.90	0.20	−0.01	−0.10	0.09	
		Medium	3.60	0.06	3.50	0.11	0.10	−0.01	0.21	
		High	5.0	0.16	4.89	0.08	0.11	−0.13	0.35	
Infant formula with probiotic^i^	*Cronobacter sakazakii* (CCUG^2^ 28863; lyophilized)	None	<0.1	NA	<0.1	NA	NA	NA	NA	0.99
		Low	1.79	0.11	1.67	0.23	0.12	−0.12	0.36	
		Medium	3.64	0.064	3.61	0.06	0.03	−0.00	0.06	
		High	4.90	0.087	4.83	0.068	0.07	−0.05	0.18	
Infant cereal with probiotic^i^	*Escherichia coli* (ATCC 25922; lyophilized)	None	<0.1	NA	<0.1	NA	NA	NA	NA	0.99
		Low	2.21	0.08	2.22	0.15	−0.01	−0.08	0.06	
		Medium	3.16	0.13	3.19	0.10	−0.03	−0.13	0.09	
		High	4.97	0.12	4.89	0.15	0.08	−0.09	0.26	
Sponge sample from stainless steel^i^	*Salmonella enterica* subsp. *enterica* Typhimurium (ATCC 14028)	None	<0.1	NA	<0.1	NA	NA	NA	NA	1.00
		Low	1.71	0.11	1.81	0.21	−0.10	−0.37	0.17	
		Medium	3.26	0.03	3.25	0.05	0.01	−0.06	0.08	
		High	4.64	0.01	4.60	0.02	0.04	0.01	0.06	
Chicken finse in n-BPW^i^	Natural	Low	1.18	0.05	1.26	0.11	−0.08	−0.18	0.03	1.00
		Medium	2.46	0.04	2.37	0.03	0.09	0.07	0.10	
		High	3.61	0.03	3.60	0.02	0.01	−0.02	0.03	
Unsalted butter	*Serratia marcescens* (ATCC 13880; 48% heat-stress injury)	None	<0.1	NA	<0.1	NA	NA	NA	NA	1.00
		Low	1.57	0.21	1.66	0.00	−0.09	−0.35	0.17	
		Medium	2.87	0.12	3.08	0.09	−0.21	−0.35	−0.05	
		High	5.54	0.10	5.46	0.20	0.08	−0.11	0.26	
Vanilla ice cream	*Klebsiella oxytoca* (ATCC 700324; 42% heat-stress injury)	None	<0.1	NA	<0.1	NA	NA	NA	NA	1.00
		Low	1.55	0.30	1.55	0.15	0.00	−0.24	0.23	
		Medium	4.93	0.05	5.04	0.04	−0.11	−0.19	−0.03	
		High	5.49	0.13	5.56	0.20	−0.07	−0.31	0.17	
Soy infant formula	*Enterobacter aerogenes* (ATCC 13048; 20% heat-stress injury)	None	<0.1	NA	<0.1	NA	NA	NA	NA	1.00
		Low	1.32	0.33	0.98	0.00	0.34	0.23	0.44	
		Medium	3.04	0.02	3.06	0.03	−0.02	−0.05	0.02	
		High	3.99	0.04	3.94	0.04	0.05	−0.01	0.12	
Chicken rinse in BPW	Natural contamination	Low	1.83	0.36	1.94	0.44	−0.11	−0.29	0.06	0.99
		Medium	2.45	0.08	2.43	0.29	0.02	−0.26	0.30	
		High	3.46	0.29	3.52	0.52	−0.06	−0.38	0.25	
Rice infant cereal	*Citrobacter freundii* (ATCC 8090; lyophilized)	None	<0.1	NA	<0.1	NA	NA	NA	NA	1.00
		Low	1.41	0.39	1.63	0.27	−0.22	−0.48	0.05	
		Medium	3.49	0.07	3.56	0.14	−0.07	−0.18	0.04	
		High	4.46	0.08	4.56	0.06	−0.11	−0.25	0.04	

a Mean of five replicate portions, plated in duplicate, after logarithmic transformation: log_10_[CFU/g (mL) + (0.1)*f*].

b Repeatability standard deviation.

c Mean difference between the candidate and reference methods.

d Confidence interval.

e 95% Lower confidence limit for difference of means.

f 95% Upper confidence limit for difference of means.

g Square of correlation coefficient.

h Independent lab performed.

i NA = Not applicable.

1 = American Type Culture Collection; 2 = Culture Collection University of Gothenburg.

Table 4 compares a singlet 24 h Peel Plate EB result to the reference method duplicate result at 48 h.

Table 5 compares a duplicate analysis of the 24 h result to the reference method.

Tables 6 and 7 present the 48 h Peel Plate EB singlet and duplicate test result compared to the reference. In all analyses, the confidence limits of the candidate method differences with the reference are within 0.5 log_10_ CFU/g (mL) and indicate no significant differences with the reference methods. Duplicate analysis compared to singlet analysis produces very little change to the mean differences or the confidence limits. The 24 h analysis statistics are comparable to 48 h analysis showing very little recovery benefit, if any, of the additional 24 h incubation. In all there were 11 matrixes studied with nine different strains of spiked EB and two with natural contamination. In every evaluation the Peel Plate EB method demonstrates equivalence to the reference methods at both the 24 h incubation and 48 h incubation times using either a singlet or duplicate analysis.

Table 8 shows cereal data in which the prepared samples were also plated on Peel Plate EBHV, 5 mL volume method. Cereal at a 1:10 dilution preparation is too thick and viscous to test with the Peel Plate method and therefore a 1:50 dilution of cereal is prescribed. This means that 5 mL of the preparation needs to be tested instead of 1 mL to obtain a CFU/0.1 g/plate result.

**Table:** Table** 8. **Peel Plate EB high-volume (HV) method (singlet count) for *Enterobacteriaceae* at 24 h versus ISO methods 21528-1 and 2

Matrix	Fortified micro- organisms (ATCC No.; % injury)	Contamination level	Candidate method		Reference method		Mean difference^c^	95% CI^e^		*r* ^2^ ^*h*^
			Mean^a^	*s*_r_ ^b^	Mean	*s*_r_		LCL^f^	UCL^g^	
Infant cereal with probiotic^i^	*Escherichia coli* (ATCC 25922; lyophilized)	None	<0.1	NA^j^	<0.1	NA	NA	NA	NA	1.00
		Low	2.23	0.13	2.22	0.15	0.01	−0.05	0.07	
		Medium	3.20	0.09	3.20	0.10	−0.00	−0.08	0.07	
		High	4.85	0.11	4.89	0.15	−0.04	−0.11	0.06	
Rice infant cereal	*Citrobacter freundii* (ATCC 8090; lyophilized)	None	<0.1	NA	<0.1	NA	NA	NA	NA	1.00
		Low	1.81	0.43	1.63	0.27	0.19	−0.12	0.49	
		Medium	3.84	0.02	3.56	0.14	0.28	0.13	0.43	
		High	4.82	0.11	4.56	0.06	0.26	0.09	0.42	
Infant cereal with probiotic^i^	*Escherichia coli* (ATCC 25922; lyophilized)	None	<0.1	NA	<0.1	NA	NA	NA	NA	1.00
		Low	2.20	0.11	2.22	0.15	−0.02	−0.12	0.10	
		Medium	3.19	0.10	3.20	0.10	−0.01	−0.06	0.07	
		High	4.98	0.12	4.89	0.15	0.08	−0.10	0.22	
Rice infant cereal	*Citrobacter freundii* (ATCC 8090; lyophilized)	None	<0.1	NA	<0.1	NA	NA	NA	NA	1.00
		Low	1.81	0.42	1.63	0.27	0.19	−0.10	0.47	
		Medium	3.83	0.04	3.56	0.14	0.27	0.11	0.43	
		High	4.85	0.10	4.56	0.06	0.29	0.14	0.44	

a Mean of five replicate portions, candidate calculated as indicated and reference plated in duplicate, after logarithmic transformation: log_10_[CFU/g (mL) + (0.1)*f*].

b Repeatability standard deviation.

c Mean difference between the candidate and reference methods.

d Confidence interval.

e 95% Lower confidence limit for difference of means.

f 95% Upper confidence limit for difference of means.

g Square of correlation coefficient.

h Independent lab performed.

i NA = Not applicable.

With the Peel Plate EB method reported in Tables 4–7, five plates were performed and the bacterial colonies on each plate summed for a CFU/0.1 g result. The Peel Plate EBHV plate is designed for 5 mL volume and, therefore, just one plate and sample addition are a preferred option of users. The Peel Plate EBHV method was not significantly different from the reference methods in these cereal evaluations. The recovery of bacteria is improved with the HV method compared to the reference method and the Peel Plate EB 1 mL method. This could reflect the fewer pipetting manipulations and faster time to pipet samples.

Table 8 shows the statistical parameters of the EBHV single plate count result at 24 h and the duplicate results at 48 h. There is no significant improvement in the recovery or the confidence limits if a 24 h single plate result is used or the 48 h duplicate result is used; both are statistically the same and equivalent to the reference methods.

## Collaborative Validation Study

The inclusivity/exclusivity and matrix studies demonstrated and satisfied validation body requirements that the candidate method accurately enumerated *Enterobacteriaceae* in select foods and environmental surfaces as claimed by the manufacturer, and that no difference in repeatability was observed between the candidate method and the reference methods. The next requirement of the harmonized MicroVal/AOAC validation is multi-laboratory collaborative study to demonstrate the candidate method can be performed by laboratories routinely doing *Enterobacteriaceae* analyses and to determine repeatability and reproducibility parameters to be assigned to the method.

### Study Design

One matrix, powdered infant formula (milk-based with iron and DHA) containing probiotic (*Lactobacillus reuteri*), was evaluated in this study. The matrix was obtained from a local retailer and screened for the presence of naturally occurring *Enterobacteriaceae* by the ISO 21528-1 reference method. No natural contamination was observed so four separate levels of contamination were targeted for the evaluation: uninoculated, 0 CFU/g (mL); low, 10–100 CFU/g (mL); medium, 100–1000 CFU/g (mL); high 1000–10 000 CFU/g (mL). To obtain the required contamination levels, bulk lots of the matrix were artificially contaminated with a lyophilized culture of *Cronobacter sakazakii* Q Laboratories (QL) isolate 17031.4 (origin–powdered infant formula) at each target contamination level. Two replicate samples from each of the four contamination levels were analyzed by both the candidate and reference methods in a paired study design by each collaborating laboratory.

A detailed collaborative study packet outlining all necessary information related to the study, including media preparation, test portion preparation, and documentation of results, was sent to each collaborating laboratory prior to the initiation of the study. A conference call was conducted prior to the initiation of the study to discuss the collaborative study packet and answer any questions from the participating laboratories.

### Preparation of the Inocula and Test Portions

The *C. sakazakii* isolate used in this evaluation was lyophilized prior to inoculation. The culture was propagated onto SBA from a Q Laboratories frozen stock culture stored at −70°C. To prepare the culture for lyophilization, a single, well-isolated colony from SBA was transferred into BHI broth and incubated at 37 ± 2°C for 18–24 h. The culture was diluted in a sterile cryoprotectant, reconstituted in 10% nonfat dry milk, and freeze dried for 48–72 h. A bulk lot of the test matrix inoculated with the culture at a high level was expected to yield all positive results. An aliquot of the high-level inoculum was further mixed with uninoculated powdered infant formula to produce the low-level inoculum. After inoculation, the matrix was held for a minimum of 2 weeks at ambient temperature (20–25°C). The inoculated test product was packaged into separate 25 g samples in sterile Whirl-Pak^®^ bags and shipped to the collaborators.

### Test Portion Distribution

All samples were labeled with a randomized, blind-coded 3-digit number affixed to the sample container. Eleven participants from ten separate locations participated. Test portions were shipped in leak-proof insulated containers via overnight delivery according to the Category B Dangerous Goods shipment regulations set forth by International Air Transport Association. Test portions were shipped at ambient temperatures (20–25°C). Upon receipt, samples were held at ambient temperature until analysis was initiated. In addition to each of the test portions, collaborators also received a test portion for each matrix labeled as ‘lactic acid bacteria’ (LAB) to determine total background count in the matrix. The LAB samples were prepared from the bulk lot of test matrix, prior to inoculation. Additionally, a temperature probe was included in the shipment. Participants were instructed to submit the data from the temperature probe upon receipt of the shipment.

### Test Portion Analysis

Collaborators followed the appropriate preparation and analysis protocol provided to them in the collaborator instructions (Version 3, February 2018). Each collaborator received eight test portions (two high, two medium, two low, and two uninoculated). Sample portions defined by ISO method, 25 g test portion was diluted with 225 mL buffered peptone water (BPW) and homogenized with a paddle blender for 2 m ± 10 s. Ten-fold serial dilutions of each sample were prepared and a 1.0 mL aliquot of each dilution was plated onto a single Peel Plate EB for each dilution. Plates were incubated at 37 ± 1°C for 24 h and enumerated. After enumeration, plates were reincubated at 37 ± 1°C for an additional 24 h (48 h total). Plates were re-enumerated at 48 h. Each spot on the plate represented an EB colony and was enumerated. Plates containing greater than 150 colonies/plate were recorded as too numerous to count. Final CFU/g (mL) results were determined by multiplying the counts by the dilution factor (reciprocal of dilution) for that plate.

Each test portion analyzed by the Peel Plate EB method was also analyzed using either the ISO 21528-1 or 21528-2 reference method in a paired study design. The uninoculated and low-level test portions were analyzed via the ISO 21528-1 reference method, and the medium- and high-level samples were analyzed via the ISO 21528-2 reference method. For ISO 21528-1, a three-tube MPN was prepared. Positive tubes, those showing turbidity indicating growth, were struck to VRBAG for visual determination of typical colonies (red to purple with or without zones of precipitate). For ISO 21528-2, serial dilutions for each sample were plated in duplicate using VRBAG. Agar plates were incubated for 24 ± 2 h at 37 ± 1°C. Typical colonies in the countable range (<150 CFU/plate) were enumerated using a standard colony counter. For both ISO 21528 Parts 1 and 2, typical colonies were confirmed positive for *Enterobacteriaceae* by a spot oxidase test and a glucose agar test.

### Statistical Analysis

Each collaborating laboratory recorded the CFU/g (mL) results for the reference methods and the candidate method on the electronic spreadsheet provided. The data sheets were submitted to the study director at the end of the study for analysis. A logarithmic_10_ transformation [CFU/g + 0.1*f*], where *f* is the reported CFU/g (mL) corresponding to the smallest reportable result]. A Youden plot was prepared to identify discrepancies between test replicates. Outliers were identified using the Cochran and Grubb’s test. The differences of means, including 95% upper and lower confidence limits, were determined for each contamination level (2, 13). If the difference of means between the two methods was between −0.5 log_10_ CFU/g (mL) and +0.5 log_10_ CFU/g (mL) it was considered that no statistical difference existed between the two methods (3, 14). The repeatability (*s*_r_) and reproducibility (*s*_R_) of the methods were also determined (5).

### Powdered Infant Formula with Probiotics

**Figure 1.**
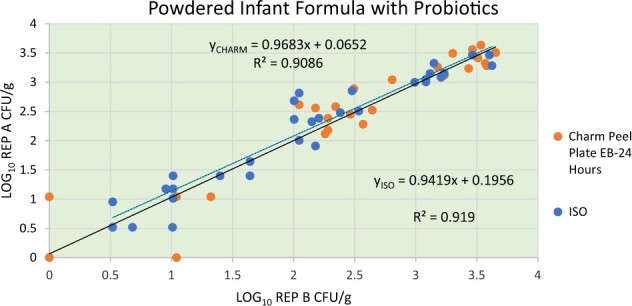
Youden’s plot for Peel Plate EB (24 h) and ISO 21528–1 and ISO 21528–2 for powdered infant formula with probiotic.

**Figure 2.**
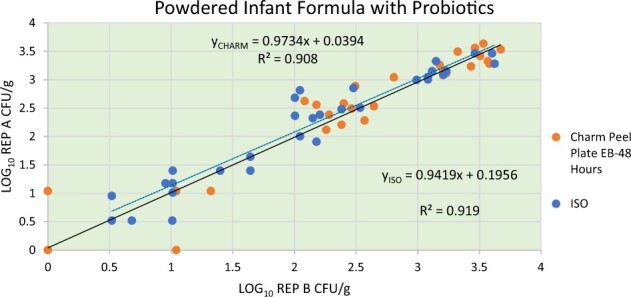
Youden’s plot for Peel Plate EB (48 h) and ISO 21528–1 and ISO 21528–2 for powdered infant formula with probiotic.

- *Results.—*Each collaborating laboratory recorded the CFU/g (mL) results for the reference methods and the candidate method on the electronic spreadsheet provided. The data sheets were submitted to the study director at the end of the study for analysis. The candidate method results at 24 and at 48 h along with the reference method results reported by each laboratory were converted to logarithmic values for statistical analysis and were plotted using a Youden’s plot. The log_10_ individual laboratory results are presented in Supplemental Appendix  Tables 1 and 2. Figures 1 and 2 present the Youden plots of each laboratory. The transformed data were analyzed for outliers by the Cochran and Grubb’s tests. No outliers were identified. The difference of means (including 95% confidence intervals), repeatability (*s*_r_), and reproducibility (*s*_R_) were determined for each contamination level. The results of the interlaboratory data analyses are presented in Table 9. In addition to the test portions, each participant that performed testing and submitted results for a LAB test, following procedures outlined in the *Compendium of Methods for the Microbiological Examination of Foods* (15), to determine the total microbial load of the test matrix. The average LAB result obtained by the collaborators was 4.1 × 10^6^ CFU/g (mL) [1.7 × 10^5^ CFU/g (mL) to 8.9 × 10^6^ CFU/g (mL)]. Supplemental Appendix  Table 3 presents the results of the LAB for each collaborator.
  1. *Peel Plate EB 24 h.—*Difference of means values [0.00, −0.16, 0.15, and 0.18 log_10_ CFU/g (mL)] for the uninoculated, low, medium, and high contamination levels indicated that no statistical significant difference existed between the candidate and reference methods. Repeatability [0.00, 0.33, 0.20, and 0.12 log_10_CFU/g (mL)] and reproducibility [0.00, 0.45, 0.26, and 0.18 log_10_CFU/g (mL)] values for each contamination level indicate that the method performed similarly within sample replicates and between laboratories throughout the range of contamination levels.
  2. *Peel Plate EB 48 h.—*Difference of means values [0.00, −0.15, 0.16, and 0.18 log_10_ CFU/g (mL)] for the uninoculated, low, medium, and high contamination levels indicated that no statistical significant difference existed between the candidate and reference method. Repeatability [0.00, 0.34, 0.25, and 0.11 log_10_ CFU/g (mL)] and reproducibility [0.00, 0.45, 0.25, and 0.17 log_10_CFU/g (mL)] values for each contamination level indicate that the method performed similarly within sample replicates and between laboratories throughout the range of contamination levels.
- *Discussion.—*No negative feedback was reported to the study directors from the ten collaborating laboratories regarding the performance of the candidate method. A few collaborators indicated that the Peel Plate EB method produced distinct colonies and were very easy to read. There were no outlier data points from any of the laboratories.

**Table 9.** Interlaboratory study results of Peel Plate EB versus ISO 21528-1 and ISO 21528-2

Peel Plate EB						ISO 21528-1 and ISO 21528-2					Difference of means^d^	Difference of means^e^ (95% LCL, UCL)
Matrix	Lot	*N* ^a^	Mean log_10_ CFU/g	*s*_r_ ^b^	*s*_R_ ^c^	Lot	*N*	Mean log_10_ CFU/g	*s*_r_	*s*_R_		
Infant formula with probiotic (24 h result)	Uninoculated	11	<0.1	NA^f^	NA	Uninoculated	11	<0.1	NA	NA	NA	NA, NA
	Low	11	0.89	0.33	0.45	Low	11	1.05	0.18	0.39	−0.16	−0.31, −0.01
	Medium	11	2.46	0.20	0.26	Medium	11	2.31	0.27	0.27	0.15	0.05, 0.25
	High	11	3.39	0.12	0.18	High	11	3.21	0.10	0.20	0.18	0.11, 0.25
Infant formula with probiotic (48 h result)	Uninoculated	11	<0.1	NA^f^	NA	Uninoculated	11	<0.1	NA^f^	NA	NA	NA, NA
	Low	11	0.90	0.34	0.45	Low	11	1.05	0.18	0.39	−0.15	−0.31, 0.1
	Medium	11	2.47	0.25	0.25	Medium	11	2.31	0.27	0.27	0.16	0.06, 0.26
	High	11	3.39	0.11	0.17	High	11	3.21	0.10	0.20	0.18	0.12, 0.25

a Number of collaborators that reported complete results.

b *s*_r_ = Repeatability.

c *s*_R_ = Reproducibility.

d Difference of the means should between −0.5 and +0.5 log_10_ CFU/g (mL).

e 95% Lower and upper confidence limits.

f NA = Not applicable.

No statistically significant difference was observed between the candidate method, at both 24 and 48 h, and the ISO reference methods when compared using the difference of means of <0.5 log_10_ CFU/g (mL). Difference of means values indicated that the candidate method produced similar results [<0.10 log_10_ CFU/g (mL)] between the 24 and 48 h incubation time points indicating that either time point is acceptable for use. Based on the data presented, the reproducibility values obtained for all contamination levels were generally similar between the candidate and reference methods, indicating that both the between- and within-laboratory variations were consistent between the candidate and reference method. These values indicate that for reproducibility, no meaningful statistical differences [absolute value of <0.50 log_10_ CFU/g (mL)] were observed in the data between the candidate and reference methods when test portions were analyzed by different analysts at each laboratory or within each sample set at a given laboratory.

### Product Consistency (Lot-to-Lot) and Stability Studies

Peel Plate EB are quality tested after manufacture following the Charm Sciences, Inc. quality control documents which are part of the quality management system have just recently been certified under the ISO 9001 (2015) system. Encompassed in the quality control evaluation are random collection of QC samples throughout the aseptic production, two tests per every 50 manufactured. These are put through a series of evaluations.

Sterility checks call for 60 tests per lot, where a lot encompasses a week’s production. Tests are rehydrated with 1 mL sterile water and incubated 72 h. There are to be no detected *Enterobacteriaceae* in any of the tests, and if one or more are detected an additional 200 tests performed with less than 1% containing one *Enterobacteriaceae* or less.

Detection and recovery evaluation (performance checks) are performed in comparison to the VRBAG reference method using comparing *n *=* *10 test pairs of various *Enterobacteriaceae* strains. Additionally, naturally EB contaminated chicken samples have been added to verify both exclusion and selection of EB with verification of detected colonies using confirmation methods. Twelve to 25 samples are compared at neat, 10^−1^, and 10^2^ dilutions to achieve a countable range 1–150 CFU/plate. Results are compared to reference methods using a statistical population analysis. Peel Plate EB population results should be within 0.2 log mean difference with a population CI greater than *P *>* *0.01.

Accelerated stress testing is performed 45 days at 37°C to assure an 18-month refrigerated shelf life and a 1-year shelf life at 0–25°C. Recovery experiments comparing *n *=* *10 test pairs of various *Enterobacteriaceae* strains are performed to verify no significant difference *P *>* *0.05 from prior production and reference methods. A non-coliform strain, *Lactobacillus*, is also evaluated to make sure there is no degradation of selection agents in the stressed tests. Real-time storage testing is also performed to verify performance at shelf date.

Additionally, production quality control specifications for the dryness of the plates, <4.5%, are reviewed and additional testing added if manufactured products exceed those specifications.

A summary of these evaluations for several lots of manufactured product are supplied in Table 10. These testing parameters are designed to assure the product consistency and stability until 1 year at 0–25°C shelf life.

**Table 10.** Quality control of three lots of Peel Plate EB

Lot no.	Date of manufacture	Sterility check (no. positive/ no. tested)	Accelerated test	Check *P*^a^ from VRBAG		One-year 25°C stress test
				VBR^b^	PL^c^	
PP-EB-009	Dec. 20, 2016	0/36	Pass	0.33	0.24	Jul. 2018
PP-EB-010	Jan. 16, 2016	0/30	Pass	0.29	0.35	Aug. 2018
PP-EB-011	Mar. 8, 2018	0/24	Pass	0.35	0.32	Oct. 2018

a *T*-test probability (*P*) of being statistically the same. Specification is >0.01 value is average of three EB strains compared.

b VBR = Reference method VBRA comparison.

c MPL = Previous Peel Plate EB comparison.

### Robustness Studies

Robustness studies were performed using perturbations of the critical steps of the Peel Plate EB method (13). The steps and perturbations evaluated were pipetting, 1.0 ± 0.1 mL; temperature of incubation, low (35) and high (39°C); and time of incubation, low (22) and high (26 h), and 46 and 48 h. The assays were performed in buffer with two ATCC strains, with ten replicate tests under each assay condition. Each perturbation condition was compared to the control condition in a paired *t*-test analysis. Results of the robustness analysis are reported in Table 11.

**Table 11.** Evaluation of Peel Plate EB assay perturbations

Assay perturbation	Bacterial strain	High and low condition	Mean CFU/mL	SD	CV%	Paired *t*-test probability of equivalence	Log difference^a^	LCL^b^	UCL^c^
Temp., °C	*Serratia marcescens* (ATCC 13880)	35	41	5	13	20	−0.06	−0.13	0.01
		39	36	7	17				
	*Citrobacter fruendii* (ATCC 8090)	35	31	5	21	54	0.05	−0.03	0.12
		39	32	8	19				
Pipet volume, µL	*Serratia marcescens* (ATCC 13880)	900	44	7	15	<0.1	0.13	0.09	0.17
		1100	60	9	14				
	*Citrobacter fruendii* (ATCC 8090)	900	48	10	20	<0.1	0.14	0.06	0.21
		1100	65	9	15				
Assay time, h	*Hafnia alvei* (ATCC 51815)	22	21	3	14	6	0.01	0.01	0.02
		50	22	3	14				
	*Enterobacter aerogenes* (ATCC 13048)	22	100	10	10	16	0.00	−0.01	0.01
		50	101	9	9				

a Log_10_ CFU/g (mL) mean difference between the low and high pairs *n *=* *10 pairs.

b 95% Lower confidence limit for difference of means.

c 95% Upper confidence limit for difference of means.

Assay temperature showed no significant difference by *t*‐test or paired log‐*t* test confidence levels >0.5. A shorter assay time did not show a significant difference by *t*‐test and there is no significant difference between the shorter (22) and longer (50 h) incubation times. Pipet volume did show a significant difference by *t*-test as would be expected with a low bias of the 900 compared to the 1100 uL dispense. Despite the measured *t*-test low bias, using the mean difference and confidence limits >0.5 log as the significance specification, the bias is not considered significant.

The effect of moisture loss from an exposed unsealed test strip and the effect of moisture loss on a test exposed for 15 min in a laminar flow hood were determined. In control experiments with sealed strips, there is less than a 1% loss of weight, while there was a 10–15% weight loss after 15 min open air exposure that would simulate an open environmental air sample taken in a food plant.Moisture loss studies were performed with three lots of EB tests in three sets (*n *=* *10 each) with Butterfield’s buffer fortified with either *Hafnia alvei* ATCC 51815*, Pantoea agglomerans* ATCC 27155, or *Enterobacter aerogenes* ATCC 13048. Plates had diluted samples added and were left exposed in a32°C incubator for 15 min to achieve volume loss of 10–15%. These were compared to a control set that were sealed and not exposed to the moisture loss step. Average and standard deviations were calculated and reported in Table 12. Overall, there were not significant differences (±2 SD) in the bacterial recovery on the control compared to the air-exposed plates on any of the three lots of tests evaluated.

**Table 12.** Effect of 15 min open air exposure of Peel Plate EB count CFU/mL

Bacterial strain	Calculations	EB Lot 011		EB Lot 012		EB lot 013	
		Control^a^	Air exposure	Control	Air exposure	Control	Air exposure
*Hafnia alvei* (ATCC 51815)	Mean	4	4	6	4	5	3
	SD	3	2	2	3	3	2
	% Change	−	0	−	−33	−	−40
*Pantoea agglomerans* (ATCC 27155)	Mean	3	5	3	3	3	3
	SD	1	2	2	2	1	1
	% Change	−	+40	−	0	−	0
*Enterobacter aerogenes* (ATCC 13048)	Mean	66	62	62	63	64	65
	SD	6	6	7	8	7	5
	% Change	−	−6	−	+3	−	+2

a Control = No air exposure.

## Conclusions

The precollaborative study demonstrates that the Peel Plate EB method incubated at 37 ± 1°C for 24 up to 48 h, without a confirmatory step, selectively detects EB and excludes non-EB. Matrix studies of heat-processed milk and dairy products, infant formula and cereals, environmental surfaces, and chicken rinse samples were not significantly different from the standard method for *Enterobacteriaceae,* ISO 21528-1:2017 Microbiology of the food chain–Horizontal method for detection and enumeration of *Enterobacteriaceae*—Part 1: Detection and Part 2: Enumeration. The candidate method was not significantly different from the reference using either a singlet plate result or duplicate plate results at both the 24 and 48 h incubation periods.

International collaborative study by 11 participants from 10 laboratories studying dried infant formula (dairy-based) containing probiotic demonstrated results not significantly different from the reference method with mean differences within −0.2 log_10_ CFU/g (mL) of ISO 21528-1 at the low concentration and within 0.2 log_10_ CFU/g (mL) of ISO 21528-2 at the medium and high concentrations. Repeatability and reproducibility values at both the 24 and 48 h were comparable to the reference methods.

The study data support that the Peel Plate EB method is equivalent to the ISO 21528 Parts 1 and 2 reference methods within heat-processed and dairy products, infant formula and cereals, stainless surfaces, and chicken carcass rinses studied.

## Recommendations

It is recommended that the Peel Plate EB be adopted as Official First Action status for the enumeration of *Enterobacteriaceae* from pasteurized whole milk, butter, nonfat dry milk, vanilla ice cream, powdered and liquid infant formula (milk-based) containing probiotic, nonprobiotic liquid infant formula (soy-based), infant rice cereal (without probiotic), infant cereal with probiotic, chicken carcass rinse with neutralized BPW, chicken carcass rinse with BPW, and stainless-steel surfaces.

## Supplemental Information

Supplemental information is available on the *J. AOAC Int*. website.

## Supplementary Material

qsaa067_Supplementary_Data
