# Supplementary material for: AOAC-OMA/MicroVal Harmonized Validation of Peel PlateTM EB (Enterobacteriaceae Bacteria), First Action 2018.05
Source: J AOAC Int. 2020 Jun 17;103(6):1588–603. doi: 10.1093/jaoacint/qsaa067 (PMC8493887; doi:10.1093/jaoacint/qsaa067)
Supplement: qsaa067_Supplementary_Data [file qsaa067_supplementary_data.docx]

**Appendix**

| **Appendix Table 1. Log_10_ *Enterobacteriaceae* counts for powdered infant formula by Peel Plate EB (24 h incubation) vs. ISO 21528-1 and ISO 21528-2** | | | | | | | | | | | | | | | | |
| --- | --- | --- | --- | --- | --- | --- | --- | --- | --- | --- | --- | --- | --- | --- | --- | --- |
| **Uninoculated** | | | | | **Low** | | | | **Medium** | | | | **High** | | | |
| Collaborator | **Peel Plate EB log_10_ CFU/g** | | **ISO 21528-1 log_10_ CFU/g** | | **Peel Plate EB log_10_ CFU/g** | | **ISO 21528-1 log_10_ CFU/g** | | **Peel Plate EB log_10_ CFU/g** | | **ISO 21528-2 log_10_ CFU/g** | | **Peel Plate EB log_10_ CFU/g** | | **ISO 21528-2 log_10_ CFU/g** | |
|  | A*^a^* | B | A | B | A | B | A | B | A | B | A | B | A | B | A | B |
| 1 | 0.00 | 0.00 | 0.00 | 0.00 | 1.041 | 1.041 | 1.176 | 1.013 | 2.179 | 2.281 | 1.908 | 2.179 | 3.117 | 3.233 | 3.149 | 3.233 |
| 2 | 0.00 | 0.00 | 0.00 | 0.00 | 0.000 | 0.000 | 0.519 | 0.519 | 3.042 | 2.807 | 2.852 | 2.479 | 3.634 | 3.533 | 3.324 | 3.149 |
| 3 | 0.00 | 0.00 | 0.00 | 0.00 | 1.041 | 1.041 | 1.176 | 0.954 | 2.281 | 2.569 | 2.507 | 2.533 | 3.417 | 3.507 | 3.004 | 3.083 |
| 4 | 0.00 | 0.00 | 0.00 | 0.00 | 1.041 | 1.322 | 1.013 | 1.013 | 2.614 | 2.045 | 2.682 | 2.004 | 3.507 | 3.654 | 3.464 | 3.603 |
| 5 | 0.00 | 0.00 | 0.00 | 0.00 | 1.041 | 1.322 | 1.398 | 1.013 | 2.558 | 2.179 | 2.382 | 2.207 | 3.558 | 3.464 | 3.464 | 3.464 |
| 6 | 0.00 | 0.00 | 0.00 | 0.00 | 1.041 | 0.000 | 0.954 | 0.519 | 2.382 | 2.281 | 2.324 | 2.149 | 3.324 | 3.569 | 3.281 | 3.624 |
| 7 | 0.00 | 0.00 | 0.00 | 0.00 | 1.041 | 1.322 | 1.643 | 1.643 | 2.117 | 2.258 | 2.004 | 2.045 | 3.149 | 3.117 | 2.996 | 2.991 |
| 8 | 0.00 | 0.00 | 0.00 | 0.00 | 1.041 | 1.041 | 0.519 | 0.681 | 2.449 | 2.464 | 2.479 | 2.382 | 3.258 | 3.179 | 3.045 | 3.083 |
| 9 | 0.00 | 0.00 | 0.00 | 0.00 | 1.041 | 1.041 | 1.398 | 1.398 | 2.887 | 2.493 | 2.364 | 2.004 | 3.281 | 3.581 | 3.083 | 3.207 |
| 10 | 0.00 | 0.00 | 0.00 | 0.00 | 0.000 | 1.041 | 0.519 | 1.009 | 2.52 | 2.644 | 2.814 | 2.045 | 3.233 | 3.433 | 3.045 | 3.083 |
| 11 | 0.00 | 0.00 | 0.00 | 0.00 | 1.041 | 1.041 | 1.398 | 1.643 | 2.581 | 2.344 | 2.364 | 2.004 | 3.493 | 3.303 | 3.149 | 3.117 |
| *^a^* A and B = Indicated duplicate test portions. | | | | | | | | | | | | | | | | |

| **Appendix Table 2. Log_10_ *Enterobacteriaceae* counts for powdered infant formula by Peel Plate EB (48 h incubation) vs. ISO 21528-1 and ISO 21528-2** | | | | | | | | | | | | | | | | |
| --- | --- | --- | --- | --- | --- | --- | --- | --- | --- | --- | --- | --- | --- | --- | --- | --- |
| **Uninoculated** | | | | | **Low** | | | | **Medium** | | | | **High** | | | |
| Collaborator | **Peel Plate EB log_10_ CFU/g** | | **ISO 21528-1 log_10_ CFU/g** | | **Peel Plate EB log_10_ CFU/g** | | **ISO 21528-1 log_10_ CFU/g** | | **Peel Plate EB log_10_ CFU/g** | | **ISO 21528-2 log_10_ CFU/g** | | **Peel Plate EB log_10_ CFU/g** | | **ISO 21528-2 log_10_ CFU/g** | |
|  | A*^a^* | B | A | B | A | B | A | B | A | B | A | B | A | B | A | B |
| 1 | 0.00 | 0.00 | 0.00 | 0.00 | 1.041 | 1.041 | 1.176 | 1.013 | 2.207 | 2.382 | 1.908 | 2.179 | 3.117 | 3.233 | 3.149 | 3.233 |
| 2 | 0.00 | 0.00 | 0.00 | 0.00 | 0.000 | 0.000 | 0.519 | 0.519 | 3.042 | 2.807 | 2.852 | 2.479 | 3.634 | 3.533 | 3.324 | 3.149 |
| 3 | 0.00 | 0.00 | 0.00 | 0.00 | 1.041 | 1.322 | 1.176 | 0.954 | 2.281 | 2.569 | 2.507 | 2.533 | 3.417 | 3.507 | 3.004 | 3.083 |
| 4 | 0.00 | 0.00 | 0.00 | 0.00 | 1.041 | 1.322 | 1.013 | 1.013 | 2.624 | 2.083 | 2.682 | 2.004 | 3.533 | 3.673 | 3.464 | 3.603 |
| 5 | 0.00 | 0.00 | 0.00 | 0.00 | 1.041 | 1.322 | 1.398 | 1.013 | 2.558 | 2.179 | 2.382 | 2.207 | 3.558 | 3.464 | 3.464 | 3.464 |
| 6 | 0.00 | 0.00 | 0.00 | 0.00 | 1.041 | 0.000 | 0.954 | 0.519 | 2.382 | 2.281 | 2.324 | 2.149 | 3.324 | 3.569 | 3.281 | 3.624 |
| 7 | 0.00 | 0.00 | 0.00 | 0.00 | 1.041 | 1.322 | 1.643 | 1.643 | 2.117 | 2.258 | 2.004 | 2.045 | 3.149 | 3.207 | 2.996 | 2.991 |
| 8 | 0.00 | 0.00 | 0.00 | 0.00 | 1.041 | 1.041 | 0.519 | 0.681 | 2.493 | 2.464 | 2.479 | 2.382 | 3.258 | 3.179 | 3.045 | 3.083 |
| 9 | 0.00 | 0.00 | 0.00 | 0.00 | 1.041 | 1.041 | 1.398 | 1.398 | 2.887 | 2.493 | 2.364 | 2.004 | 3.281 | 3.581 | 3.083 | 3.207 |
| 10 | 0.00 | 0.00 | 0.00 | 0.00 | 0.000 | 1.041 | 0.519 | 1.009 | 2.533 | 2.644 | 2.814 | 2.045 | 3.233 | 3.433 | 3.045 | 3.083 |
| 11 | 0.00 | 0.00 | 0.00 | 0.00 | 1.041 | 1.041 | 1.398 | 1.643 | 2.581 | 2.4 | 2.364 | 2.004 | 3.493 | 3.324 | 3.149 | 3.117 |
| *^a^* A and B = Indicated duplicate test portions. | | | | | | | | | | | | | | | | |

**Appendix Table 3. Results of lactic acid bacteria for collaborating laboratories**

| Lab | Powdered infant formula with probiotics  (CFU/g)*^a^* |
| --- | --- |
| 1 | 3.6 x 10^6^ |
| 2 | 4.6 x 10^5^ |
| 3 | 4.7 x 10^6^ |
| 4 | 6.1 x 10^6^ |
| 5 | 8.9 x 10^6^ |
| 6 | 5.2 x 10^6^ |
| 7 | 4.8 x 10^5^ |
| 8 | 1.7 x 10^5^ |
| 9 | 8.6 x 10^6^ |
| 10 | 5.2 x 10^6^ |
| 11 | 2.1 x 10^6^ |

*^a^* Samples analyzed by CMMEF Chapter 20.
